# Association between *MnSOD* Val16Ala Polymorphism and Cancer Risk: Evidence from 33,098 Cases and 37,831 Controls

**DOI:** 10.1155/2018/3061974

**Published:** 2018-09-02

**Authors:** Ping Wang, Yanfeng Zhu, Shoumin Xi, Sanqiang Li, Yanle Zhang

**Affiliations:** ^1^Department of Biochemistry and Molecular Biology, Medical College, Henan University of Science and Technology, Luoyang, Henan 471023, China; ^2^School of Materials Science and Engineering, Henan University of Science and Technology, Luoyang, Henan 471023, China

## Abstract

Manganese superoxide dismutase (MnSOD) plays a critical role in the defense against reactive oxygen species. The association between *MnSOD* Val16Ala polymorphism and cancer risk has been widely studied, but the results are contradictory. To obtain more precision on the association, we performed the current meta-analysis with 33,098 cases and 37,831 controls from 88 studies retrieved from PubMed, Embase, Chinese National Knowledge Infrastructure (CNKI), and Wanfang databases. Pooled odds ratios (ORs) and 95% confidence intervals (CIs) were used to assess the strength of association. We found that the polymorphism was associated with an increased overall cancer risk (homozygous: OR = 1.09, 95% CI = 1.00–1.19; heterozygous: OR = 1.07, 95% CI = 1.02–1.12; dominant: OR = 1.08, 95% CI = 1.02–1.14; and allele comparison: OR = 1.06, 95% CI = 1.02–1.11). Stratification analysis further showed an increased risk for prostate cancer, Asians, Caucasians, population-based studies, hospital-based studies, low quality and high quality studies. However, the increased risk for *MnSOD* Val16Ala polymorphism among Asians needs further validation based on the false-positive report probability (FPRP) test. To summarize, this meta-analysis suggests that the *MnSOD* Val16Ala polymorphism is associated with significantly increased cancer risk, which needs further validation in single large studies.

## 1. Introduction

Cancer is one of the leading causes of death across the world, with an estimate of over 20 million new cancer cases that will occur per year as early as 2025 [1]. Although great efforts have been devoted to cancer treatment, cancer still poses a huge threat to human health. Carcinogenesis is rather complex, and mounting evidence suggests that reactive oxygen species- (ROS-) related oxidative damage is involved in this process [2–4].

Among the endogenous antioxidants, manganese superoxide dismutase (MnSOD) is one of the critical enzymes which defends against ROS in the mitochondria. The *MnSOD* gene, located on chromosome 6q25.3, is composed of four introns and five extrons. Currently, several single-nucleotide polymorphisms (SNPs) in the *MnSOD* gene have been reported, of which the most extensively studied one is Val16Ala. Since this residue is 9 amino acids upstream of the cleavage site, it has also been called Val9Ala (rs4880) polymorphism [5]. A previous study has shown that Ala-*MnSOD* allowed more efficient *MnSOD* localized to the mitochondria than the Val-variant form [6]. In view of this, it is speculated that the Val form of *MnSOD* may be associated with higher levels of ROS and increased susceptibility to cancer.

Several studies have found the associations between the Val form of the *MnSOD* gene and increased cancer risk [7–9], but a majority of studies showed the Ala form to be associated with higher cancer risk, such as breast cancer [10, 11], esophageal cancer [12], colorectal cancer [13], and cervical cancer [14], and some other studies find no significant association between this polymorphism and cancer risk [15–18]. To draw a more comprehensive estimation of this possible association, we conducted the present meta-analysis to evaluate the relevance of this variant with susceptibility of cancer.

## 2. Materials and Methods

### 2.1. Search Strategy

We systematically searched the PubMed, Embase, Chinese National Knowledge Infrastructure (CNKI), and Wanfang databases for all related publications using the following keywords: “*MnSOD* or manganese superoxide dismutase,” “polymorphism or variant or variation,” and “cancer or carcinoma or tumor or neoplasm” (the last search was updated on February 22, 2018). Additional relevant studies were searched manually from the references or review articles about this topic. If studies had overlapped data, only the one with the most participants was included in this analysis.

### 2.2. Inclusion and Exclusion Criteria

The inclusion criteria were as follows: (1) case-control studies, (2) studies assessing the association between *MnSOD* Val16Ala polymorphism and cancer risk, (3) and provision of detailed data about genotype and allele distribution of the studied polymorphism. Studies were excluded if any of the following aspects existed: (1) duplicate publications, (2) review articles or meta-analyses, (3) not a case-control study, and (4) genotype frequencies in the control departure from Hardy-Weinberg equilibrium (HWE).

### 2.3. Data Extraction

Two authors (Ping Wang and Yanfeng Zhu) independently extracted the data from included studies according to the criteria mentioned above. Disagreement was resolved by discussion until a consensus was reached. The following information was collected from each study: first author's surname, year of publication, country of origin, ethnicity, cancer type, control source (hospital-based or population-based), genotyping methods, and numbers of cases and controls with the Val/Val, Val/Ala, and Ala/Ala genotypes.

### 2.4. Quality Assessment

The quality of each included study was assessed independently by two authors using the criteria from a previous study [19]. Quality scores were rated from 0 to 15, and the studies were classified as high-quality studies (scores > 9) and low-quality studies (scores ≤ 9).

### 2.5. Statistical Analysis

The strength of association between the *MnSOD* Val16Ala polymorphism and cancer risk was assessed by calculating the odd ratios (ORs) with the corresponding 95% confidence intervals (CIs). The pooled ORs of five comparison models were calculated: homozygous model (Ala/Ala versus Val/Val), heterozygous model (Val/Ala versus Val/Val), recessive model [Ala/Ala versus (Val/Val + Val/Ala)], dominant model [(Ala/Ala + Val/Ala) versus Val/Val], and an allele comparison (Ala versus Val). We used the chi-square-based *Q* test to check the between-study heterogeneity, and the fixed-effects model (the Mantel-Haenszel method) [20] was used when no significant heterogeneity was found (*P* > 0.1). Otherwise, the random-effects model (the Dersimonian and Laird method) [21] was applied. The stratification analysis was performed by cancer type (cancer types with less than three studies would be merged into the “others” group), ethnicity (Asians, Caucasians, Africans, or mixed which contained more than one ethnic group), control source (hospital-based studies and population-based studies), and quality scores (≤9 and >9). Publication bias was examined using Begg's funnel plot [22] and Egger's linear regression test [23]. Sensitivity analysis was carried out to assess the results stability by excluding one study each time and revaluating the pooled ORs and 95% CIs.

The false-positive report probability (FPRP) was calculated for all the significant findings in the present study. We set 0.2 as a FPRP threshold and assign a prior probability of 0.1 to detect an OR of 0.67/1.50 (protective/risk effects) for an association with the genotypes under investigation [24, 25]. FPRP values less than 0.2 were considered as noteworthy associations. All the statistical tests were performed with STATA software (version 12.0; Stata Corporation, College Station, TX). Two-sided *P* values <0.05 were considered statistically significant.

## 3. Results

### 3.1. Study Characteristics

**Figure 1:**
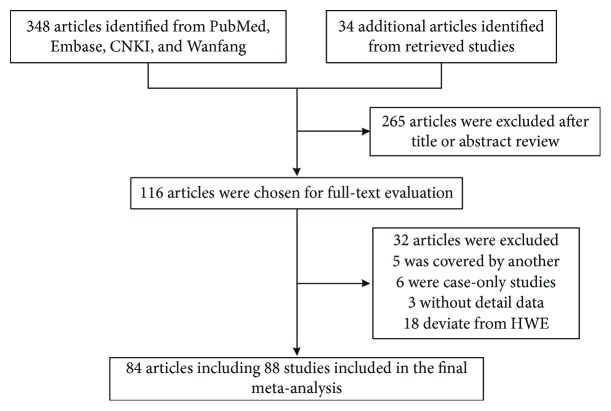
Flowchart of included studies for the association between *MnSOD* Val16Ala polymorphism and cancer susceptibility.

As shown in Figure 1, a total of 348 articles were identified from PubMed, Embase, CNKI, and Wanfang databases, and 34 more articles were identified by reading the references of retrieved publications. After reading the titles and abstracts, 266 articles were excluded, leaving 116 articles for further assessment. Among them, six were excluded as case-only studies [26–31], five [32–36] were covered by other included publications [7, 37, 38], three were without detailed data for further analysis [39–41], and 18 deviated from HWE [42–59]. Finally, a total of 84 case-control publications [7–18, 37, 38, 60–129] were included in this meta-analysis. Of the 84 publications, three publications [37, 69, 82] with two ethnic groups were considered as two independent studies and one publication [119] with two cancer types were also considered as two independent studies.

For the two studies in the publication [119] with the same control group, the number of control was only calculated once in the total number. Overall, 88 studies with 33,098 cases and 37,831 controls were included in this meta-analysis. Of the 88 studies, 24 studies focused on breast cancer [9–11, 16, 38, 60, 61, 68, 69, 71, 72, 77, 88, 93, 96, 97, 100, 105, 109, 114, 119, 122, 127]; 17 on prostate cancer [37, 66, 74, 79, 82, 85, 86, 89, 95, 106, 111, 113, 120, 125, 128]; six for each of the following cancer types, such as lung cancer [7, 17, 18, 65, 92, 118], bladder cancer [8, 15, 67, 75, 112, 117], and pancreatic cancer [64, 91, 102, 107, 108, 121]; five on colorectal cancer [13, 63, 73, 94, 101]; three for each of the following cancer types, such as ovarian cancer [70, 81, 87], hepatocellular carcinoma [98, 99, 129], and non-Hodgkin's lymphoma [76, 78, 110]; and the other with fewer than three studies for each cancer type. Of all the studies, 56 studies were performed on Caucasians, 18 studies on Asians, and seven studies on Africans and mixed ethnicity, respectively. When classified by source of control, 48 were population-based and 40 were hospital-based. In addition, according to the quality score, 49 studies were considered as high-quality and 39 studies were considered as low-quality. The characteristics of the included studies are shown in Table 1.

### 3.2. Meta-Analysis Results

**Figure 2:**
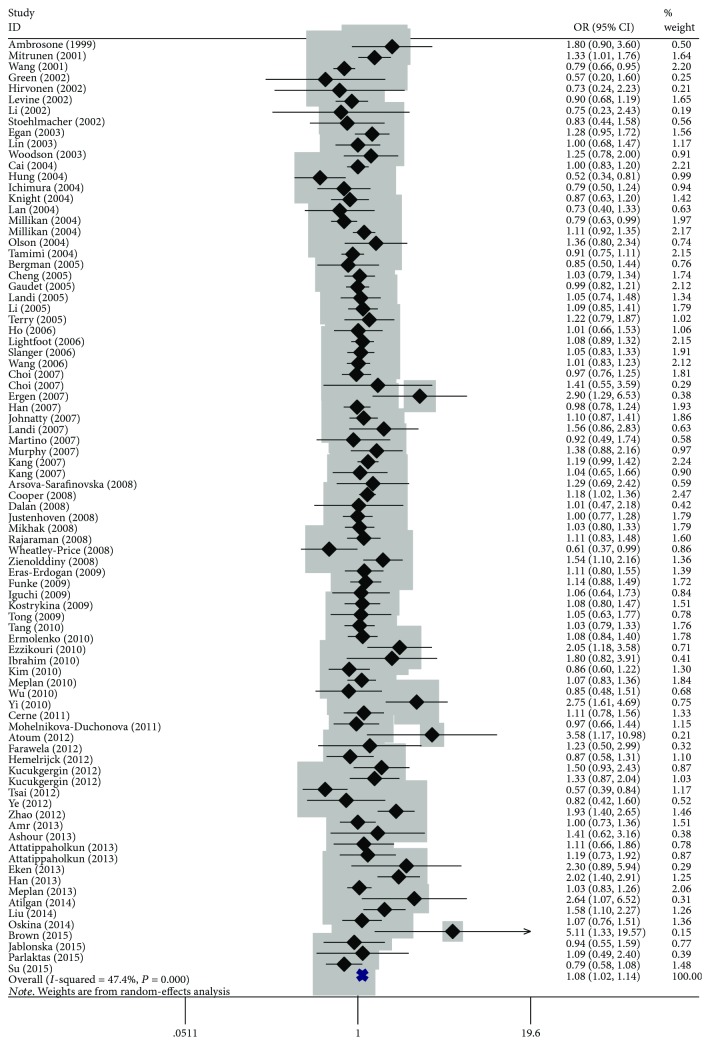
Forest plot of overall cancer risk associated with *MnSOD* Val16Ala polymorphism by dominant model. For each study, the estimated of OR and its 95% CI are plotted with a box and a horizontal line. ◇, pooled ORs and its 95% CIs.

The overall results suggested there was a significant association between *MnSOD* Val16Ala polymorphism and cancer risk (homozygous: OR = 1.09, 95% CI = 1.00–1.19, *P* < 0.001; heterozygous: OR = 1.07, 95% CI = 1.02–1.12, *P* = 0.001; dominant: OR = 1.08, 95% CI = 1.02–1.14, *P* < 0.001; and allele comparison: OR = 1.06, 95% CI = 1.02–1.11, *P* < 0.001) (Table 2, Figure 2). In the subgroup analysis, a statistically significant association was found for prostate cancer (heterozygous: OR = 1.14, 95% CI = 1.05–1.24, *P* = 0.765; dominant: OR = 1.14, 95% CI = 1.05–1.23, *P* = 0.552; and allele comparison: OR = 1.07, 95% CI = 1.00–1.15, *P* = 0.106), Asians (homozygous: OR = 1.82, 95% CI = 1.15–2.88, *P* = 0.020, and recessive: OR = 1.76, 95% CI = 1.16–2.68, *P* = 0.065), Caucasians (heterozygous: OR = 1.08, 95% CI = 1.03–1.13, *P* = 0.208; dominant: OR = 1.08, 95% CI = 1.02–1.14, *P* = 0.011; and allele comparison: OR = 1.04, 95% CI = 1.00–1.09, *P* < 0.001), population-based studies (homozygous: OR = 1.10, 95% CI = 1.01–1.19, *P* < 0.001; heterozygous: OR = 1.07, 95% CI = 1.02–1.12, *P* = 0.263; dominant: OR = 1.07, 95% CI = 1.02–1.13, *P* = 0.071; and allele comparison: OR = 1.04, 95% CI = 1.00–1.08, *P* = 0.006), hospital-based studies (recessive: OR = 1.16, 95% CI = 1.01–1.34, *P* < 0.001, and allele comparison: OR = 1.13, 95% CI = 1.03–1.24, *P* < 0.001), low-quality studies (allele comparison: OR = 1.12, 95% CI = 1.02–1.23, *P* < 0.001) and high-quality studies (homozygous: OR = 1.08, 95% CI = 1.00–1.17, *P* = 0.001; heterozygous: OR = 1.07, 95% CI = 1.02–1.13, *P* = 0.067; dominant: OR = 1.07, 95% CI = 1.02–1.14, *P* = 0.002; and allele comparison: OR = 1.04, 95% CI = 1.00–1.09, *P* < 0.001).

### 3.3. Heterogeneity and Sensitivity Analysis

As shown in Table 2, substantial heterogeneities were found among all studies for the *MnSOD* Val16Ala polymorphism and overall cancer risk (homozygous: *P* < 0.001; heterozygous: *P* = 0.001; recessive: *P* < 0.001; dominant: *P* < 0.001; and allele comparison: *P* < 0.001). Therefore, the random-effects model was used to generate wider CIs. The leave-one-out sensitivity analysis indicated that no single study could change the pooled ORs obviously (data not shown).

### 3.4. Publication Bias

Begg's funnel plot and Egger's test were performed to evaluate the publication bias of 88 studies, and we found significant publication bias for the homozygous model (*P* = 0.049), recessive model (*P* = 0.007), dominant model (*P* = 0.042), and allele comparison (*P* = 0.007), but not for the heterozygous model (*P* = 0.056). Therefore, the Duval and Tweedie nonparametric “trim and fill” method was used to adjust for publication bias. The “trim and fill” method did not draw different conclusions (data not shown), indicating that our findings were statistically robust.

### 3.5. False-Positive Report Probability (FPRP) Analysis

The FPRP values were calculated for all the significant findings (Table 3). With the assumption of a prior probability of 0.1, the FPRP results revealed that three genetic models [Val/Ala versus Val/Val, (Ala/Ala + Val/Ala) versus Val/Val, and Ala versus Val] of the *MnSOD* Val16Ala polymorphism were truly associated with increased cancer risk (FPRP = 0.032, 0.045, and 0.106, resp.). In addition, according to the FPRP results, we confirmed that the *MnSOD* Val16Ala polymorphism was associated with cancer risk for prostate cancer (heterozygous: FPRP = 0.020 and dominant: FPRP = 0.006), Caucasians (heterozygous: FPRP = 0.008 and dominant: FPRP = 0.045), population-based studies (homozygous: FPRP = 0.136, heterozygous: FPRP = 0.032 and dominant: FPRP = 0.119), hospital-based studies (allele comparison: FPRP = 0.082), low-quality studies (allele comparison: FPRP = 0.138), and high-quality studies (heterozygous: FPRP = 0.119).

## 4. Discussion

In this meta-analysis, we comprehensively assessed the association between *MnSOD* Val16Ala polymorphism and cancer risk through 88 studies, and we found that this gene polymorphism was significantly associated with overall cancer risk. Further, stratification analysis revealed that the association was more obvious for risk of prostate cancer, Asians, Caucasians, population-based studies, hospital-based studies, low-quality studies, and high-quality studies. To avoid the false-positive results of the meta-analysis, we performed the FPRP analysis for the significant findings by setting as the prior probability of 0.1, and the results suggested that the association between *MnSOD* Val16Ala polymorphism and cancer risk for Asians was false positive, which may due to limited sample size.

MnSOD is a mitochondrial enzyme that converts superoxide radical O_2_^−^ into H_2_O_2_, and it plays a critical role in human cells. Studies have revealed that the aberrant expression of MnSOD is involved in many types of cancers. Our current study indicated that the *MnSOD* Val16Ala polymorphism was significantly associated with an increased overall cancer risk. Previous meta-analyses have also assessed the association of *MnSOD* Val16Ala polymorphism with cancer susceptibility. The study carried out by Kang [130] analyzed *MnSOD* Val16Ala polymorphism and cancer risk, consisting 52 studies with 26,865 cases and 32,464 controls, in which no significant association was found between this polymorphism and overall cancer risk. In the subgroup analysis, statistically significant associations were found between this polymorphism and non-Hodgkin lymphoma, lung cancer, and colorectal cancer. Another meta-analysis [131] including 7366 cases and 9102 controls found no overall association of *MnSOD* Val16Ala polymorphism for cancer risk. Some of the significant associations detected in the previous meta-analyses were not found in the present study; for example, *MnSOD* Val16Ala polymorphism was associated with the risk of hepatocellular carcinoma [132, 133], esophageal cancer [134], and lung cancer [134]. The discrepancy that occurred may be because our current study was based on a much larger sample size, allowing the more precise detection of the association. In the subgroup analysis by cancer type, we found a significant association between *MnSOD* Val16Ala polymorphism and elevated prostate cancer risk, and no significant association between this polymorphism and breast cancer, which were consistent with previous meta-analyses [131, 134–137].

In spite of genetic importance, environment factors such as dietary pattern and exercise play important roles in the development of cancer. Recently, several studies have investigated the association between dietary intake of antioxidant-rich foods and *MnSOD* Val16Ala polymorphism in breast cancer [60], prostate cancer [89], and cervical cancer [14]. Despite the lack of consistent data, the results suggested that the *MnSOD* Val16Ala polymorphism and cancer risk could be modulated by dietary factors. Besides, a previous study had shown that moderate exercise training is beneficial for prostate cancer [138], and evidence showed that exercise training may result in positive MnSOD modulation through redox sensitive pathways [139].

The current meta-analysis has several advantages. First, we included the latest publications in the present study and also the publications written in Chinese. Second, the quality of included studies was assessed by the quality score criteria. Third, the FPRP test was performed to make the results more trustworthy and robust. Although the study is the largest and most comprehensive one regarding the association between *MnSOD* Val16Ala polymorphism and all cancer types, there were still some limitations that should be addressed. First, the number of cases in each study was small (<1000) in all but seven studies [11, 38, 69, 78, 82, 86, 119], which may have an effect on the investigation of the real association. Second, the results were based on unadjusted estimates, which might make the results imprecise. Third, only publications in English and Chinese were included, which could lead to selection bias. Fourth, in the subgroup analysis by cancer type, less than three studies were included for some types of cancer, which may affect the detection of the real association. Finally, the potential gene-gene, and gene-environment interactions were not investigated due to the lack of original information.

Despite of these limitations, this meta-analysis indicated there was a significant association between *MnSOD* Val16Ala polymorphism and cancer risk, which should be further validated by single large studies.

## Figures and Tables

**Table 1:** Characteristics of studies included in the meta-analysis.

Surname (ref)	Year	Country	Ethnicity	Cancer type	Control source	Genotype method	Case				Control				MAF	HWE	Score
							Val/Val	Val/Ala	Ala/Ala	All	Val/Val	Val/Ala	Ala/Ala	All			
Ambrosone et al. [60]	1999	USA	Caucasian	Breast	PB	PCR-RFLP	16	53	45	114	25	62	23	110	0.49	0.181	12
Mitrunen et al. [10]	2001	Finland	Caucasian	Breast	PB	PCR-RFLP	124	255	100	479	153	231	98	482	0.44	0.526	13
Wang et al. [7]	2001	USA	Caucasian	Lung	HB	Pyrosequencing	305	551	245	1101	288	628	323	1239	0.49	0.609	9
Green et al. [61]	2002	UK	Caucasian	Breast	HB	PCR-RFLP	13	17	9	39	8	22	6	36	0.47	0.175	5
Hirvonen et al. [62]	2002	Finland	Caucasian	MPM	PB	PCR-RFLP	6	11	3	20	15	36	12	63	0.48	0.248	9
Levine et al. [63]	2002	USA	Mixed	CRC	PB	PCR-RFLP	139	209	108	456	140	234	121	495	0.48	0.237	12
Li et al. [64]	2002	USA	Caucasian	Pancreatic	PB	PCR-RFLP	10	11	3	24	8	10	5	23	0.43	0.580	6
Stoehlmacher et al. [13]	2002	USA	Caucasian	CRC	PB	TaqMan	25	65	35	125	21	64	37	122	0.43	0.456	5
Egan et al. [16]	2003	USA	Caucasian	Breast	PB	PCR-RFLP	102	250	118	470	130	240	127	497	0.50	0.446	10
Lin et al. [65]^a^	2003	China	Asian	Lung	HB	PCR-RFLP	139	59 (Val/Ala + Ala/Ala)		198	233	99 (Val/Ala + Ala/Ala)		332	NA	NA	10
Woodson et al. [66]	2003	USA	Caucasian	Prostate	PB	MALDI-TOF MS	43	98	58	199	49	102	40	191	0.48	0.330	12
Cai et al. [11]	2004	China	Asian	Breast	PB	PCR-RFLP	831	266	28	1125	884	290	23	1197	0.14	0.890	15
Hung et al. [8]	2004	Italy	Caucasian	Bladder	HB	PCR-RFLP	68	89	44	201	45	115	54	214	0.48	0.262	9
Ichimura et al. [67]	2004	Japan	Asian	Bladder	HB	PCR-RFLP	169	41	3	213	157	48	4	209	0.13	0.882	11
Knight et al. [68]	2004	Canada	Caucasian	Breast	PB	PCR-SSCP	107	187	105	399	90	195	87	372	0.50	0.350	14
Lan et al. [17]	2004	China	Asian	Lung	PB	Real-time PCR	93	23	3	119	81	30	1	112	0.14	0.321	10
Millikan et al. [69]	2004	USA	African	Breast	PB	TaqMan	259	372	129	760	196	357	124	677	0.45	0.083	13
Millikan et al. [69]	2004	USA	Caucasian	Breast	PB	TaqMan	273	681	311	1265	266	586	283	1135	0.49	0.269	13
Olson et al. [70]	2004	USA	Caucasian	Ovarian	HB	MALDI-TOF MS	27	64	27	118	51	87	39	177	0.47	0.869	9
Tamimi et al. [71]	2004	USA	Caucasian	Breast	PB	Mixed^d^	255	468	245	968	297	612	296	1205	0.50	0.584	15
Bergman et al. [9]	2005	Sweden	Caucasian	Breast	PB	Sequencing	33	73	12	118	43	88	43	174	0.50	0.879	11
Cheng et al. [72]	2005	China	Asian	Breast	HB	MassARRAY	343	115	11	469	545	183	11	739	0.14	0.322	11
Gaudet et al. [38]	2005	USA	Caucasian	Breast	PB	MALDI-TOF MS	253	511	270	1034	264	539	281	1084	0.49	0.862	14
Landi et al. [73]	2005	Spain	Caucasian	CRC	HB	APEX	94	164	77	335	88	151	64	303	0.46	0.958	5
Li et al. [74]	2005	USA	Caucasian	Prostate	PB	PCR-RFLP	132	288	147	567	190	379	195	764	0.50	0.829	14
Terry et al. [75]	2005	USA	Caucasian	Bladder	HB	MALDI-TOF MS	54	122	59	235	57	103	54	214	0.49	0.586	8
Ho et al. [18]^c^	2006	China	Asian	Lung	HB	PCR-RFLP	176	58	0	234	180	52	7	239	0.14	0.184	7
Lightfoot et al. [76]	2006	USA and UK	Caucasian	NHL	PB	TaqMan	211	463	229	903	358	713	371	1442	0.50	0.676	13
Slanger et al. [77]	2006	Germany	Caucasian	Breast	PB	TaqMan	144	318	152	614	263	528	289	1080	0.49	0.477	14
Wang et al. [78]	2006	USA	Mixed	NHL	PB	TaqMan	285	545	290	1120	240	486	211	937	0.48	0.240	13
Cengiz et al. [15]^b^	2007	Turkey	Caucasian	Bladder	HB	PCR-RFLP	34 (Val/Val + Val/Ala)		17	51	34 (Val/Val + Val/Ala)		19	53	NA	NA	7
Choi et al. [37]	2007	USA	Caucasian	Prostate	PB	MALDI-TOF MS	112	239	104	455	293	610	311	1214	0.49	0.857	13
Choi et al. [37]	2007	USA	African	Prostate	PB	MALDI-TOF MS	7	15	6	28	39	52	31	122	0.47	0.112	10
Ergen et al. [79]^c^	2007	Turkey	Caucasian	Prostate	HB	PCR-RFLP	19	25	6	50	32	18	0	50	0.18	0.121	7
Han et al. [80]	2007	USA	Caucasian	Skin	PB	TaqMan	184	402	187	773	196	425	212	833	0.49	0.549	15
Johnatty et al. [81]	2007	Australia	Caucasian	Ovarian	PB	Real-time PCR	123	273	147	543	276	546	308	1130	0.49	0.269	11
Kang et al. [82]	2007	USA	Caucasian	Prostate	PB	TaqMan	275	578	297	1150	376	686	320	1382	0.48	0.835	13
Kang et al. [82]	2007	USA	African	Prostate	PB	TaqMan	31	57	15	103	122	194	79	395	0.45	0.906	11
Landi et al. [83]	2007	Italy	Caucasian	MPM	HB	APEX	16	27	37	80	98	170	81	349	0.48	0.661	9
di Martino et al. [84]	2007	USA	Caucasian	Esophageal	HB	PCR-RFLP	32	73	35	140	20	39	34	93	0.42	0.171	8
Murphy et al. [12]	2007	Ireland	Caucasian	Esophageal	PB	SNaPshot	44	103	60	207	60	113	48	221	0.47	0.703	11
Arsova-Sarafinovska et al. [85]	2008	Turkey	Caucasian	Prostate	HB	Real-time PCR	19	46	20	85	41	73	37	151	0.49	0.690	9
Cooper et al. [86]	2008	USA	Caucasian	Prostate	PB	TaqMan	602	1352	680	2634	423	789	424	1636	0.50	0.152	15
Dalan et al. [87]	2008	Turkey	Caucasian	Ovarian	PB	PCR-RFLP	30	19	6	55	28	17	6	51	0.28	0.196	7
Justenhoven et al. [88]	2008	Germany	Caucasian	Breast	PB	MALDI-TOF MS	159	312	133	604	163	313	145	621	0.49	0.824	14
Mikhak et al. [89]	2008	USA	Caucasian	Prostate	PB	TaqMan	156	320	166	642	162	331	159	652	0.50	0.695	14
Rajaraman et al. [90]	2008	USA	Caucasian	Brain	HB	TaqMan	129	262	123	514	122	220	109	451	0.49	0.617	10
Wheatley-Price et al. [91]	2008	USA	Caucasian	Pancreatic	HB	TaqMan	33	58	31	122	61	165	105	331	0.43	0.786	11
Zienolddiny et al. [92]	2008	Norway	Caucasian	Lung	PB	APEX	74	175	70	319	119	178	78	375	0.45	0.448	12
Eras-Erdogan et al. [93]	2009	Turkey	Caucasian	Breast	PB	PCR-RFLP	107	113	30	250	150	141	39	330	0.33	0.508	8
Funke et al. [94]	2009	Germany	Caucasian	CRC	PB	Pyrosequencing	136	321	166	623	146	294	163	603	0.49	0.554	12
Iguchi et al. [95]	2009	USA	Mixed	Prostate	HB	PCR-RFLP	41	86	60	187	40	96	39	175	0.50	0.199	6
Kostrykina et al. [96]	2009	Russia	Caucasian	Breast	PB	TaqMan	123	233	119	475	103	183	90	376	0.48	0.622	12
Tong et al. [14]^a^	2009	Korea	Asian	Cervical	HB	SNaPshot	72	27 (Val/Ala + Ala/Ala)		99	194	69 (Val/Ala + Ala/Ala)		263	NA	NA	7
Ermolenko et al. [97]	2010	Russia	Caucasian	Breast	HB	Real-time PCR	228	454	239	921	121	235	104	460	0.48	0.620	9
Ezzikouri et al. [98]	2010	Morocco	Caucasian	HCC	PB	PCR-RFLP	21	45	30	96	81	101	40	222	0.41	0.388	11
Ibrahim et al. [99]	2010	Egypt	African	HCC	HB	PCR-RFLP	16	32	27	75	19	28	11	58	0.43	0.904	8
Kim et al. [100]	2010	Korea	Asian	Breast	HB	TaqMan	234	66	4	304	279	90	7	376	0.14	0.934	11
Méplan et al. [101]	2010	Czech	Caucasian	CRC	HB	AS-PCR	172	358	189	719	165	318	174	657	0.49	0.415	9
Tang et al. [102]	2010	USA	Mixed	Pancreatic	HB	TaqMan	143	278	137	558	167	309	162	638	0.50	0.429	11
Wu et al. [103]	2010	China	Asian	Oral	HB	Real-time PCR	91	28	2	121	88	32	2	122	0.15	0.637	9
Yi et al. [104]	2010	China	Asian	Gastric	HB	SNaPshot	85	48	7	140	119	27	1	147	0.10	0.690	9
Cerne et al. [105]	2011	Slovenia	Caucasian	Breast	HB	TaqMan	118	269	143	530	65	134	71	270	0.51	0.910	8
Cheng et al. [106]^b^	2011	USA	Mixed	Prostate	PB	MALDI-TOF MS	152 (Val/Val + Val/Ala)		50	202	1054 (Val/Val + Val/Ala)		374	1428	NA	NA	13
Mohelnikova-Duchonova et al. [107]	2011	Czech	Caucasian	Pancreatic	PB	Real-time PCR	66	121	48	235	73	134	58	265	0.47	0.812	10
Zhang et al. [108]^b^	2011	USA	Mixed	Pancreatic	PB	TaqMan	129 (Val/Val + Val/Ala)		60	189	365 (Val/Val + Val/Ala)		121	486	NA	NA	13
Atoum et al. [109]^c^	2012	Jordan	Caucasian	Breast	HB	PCR-RFLP	22	43	0	65	11	6	0	17	0.18	0.377	6
Farawela et al. [110]	2012	Egypt	African	NHL	PB	PCR-RFLP	10	50	40	100	12	49	39	100	0.37	0.568	9
Hemelrijck et al. [111]	2012	Germany	Caucasian	Prostate	PB	MassARRAY	50	100	53	203	80	190	90	360	0.49	0.285	13
Kucukgergin et al. [112]	2012	Turkey	Caucasian	Bladder	HB	PCR-RFLP	52	68	37	157	89	99	36	224	0.38	0.341	8
Kucukgergin et al. [113]	2012	Turkey	Caucasian	Prostate	HB	PCR-RFLP	43	65	26	134	66	69	24	159	0.37	0.398	8
Tsai et al. [114]^a^	2012	China	Asian	Breast	HB	Real-time PCR	192	68 (Val/Ala + Ala/Ala)		260	138	86 (Val/Ala + Ala/Ala)		224	NA	NA	8
Ye et al. [115]	2012	China	Asian	NPC	HB	PCR	88	15	2	105	110	23	3	136	0.11	0.191	8
Zhao et al. [116]	2012	China	Asian	Brain	HB	OpenArray	241	107	31	379	293	81	6	380	0.12	0.882	11
Amr et al. [117]	2013	Egypt	African	Bladder	PB	TaqMan	127	188	99	414	109	160	87	356	0.47	0.065	13
Ashour et al. [118]	2013	Egypt	African	Lung	PB	TaqMan	17	27	6	50	21	25	4	50	0.33	0.355	9
Attatippaholkun and Wikainapakul [119]	2013	Thailand	Asian	Cervical	HB	SNaPshot	64	39	4	107	84	48	3	135	0.20	0.184	7
Attatippaholkun et al. [119]	2013	Thailand	Asian	Breast	HB	SNaPshot	82	54	5	141	84	48	3	135	0.20	0.184	7
Eken et al. [120]	2013	Turkey	Caucasian	Prostate	HB	Real-time PCR	7	17	9	33	31	37	13	81	0.39	0.726	8
Han et al. [121]	2013	Korea	Asian	Pancreatic	PB	PCR-SSCP	190	85	19	294	236	59	5	300	0.12	0.558	12
Méplan et al. [122]	2013	Denmark	Caucasian	Breast	PB	TaqMan	228	485	226	939	237	494	227	958	0.49	0.331	14
Atilgan et al. [123]	2014	Turkey	Caucasian	RCC	HB	Probe	10	17	14	41	23	19	8	50	0.35	0.244	5
Liu et al [124]	2014	China	Asian	OSCC	HB	PCR-RFLP	272	83	7	362	296	61	1	358	0.09	0.243	10
Oskina et al. [125]	2014	Russia	Caucasian	Prostate	PB	TaqMan	92	194	94	380	86	152	99	337	0.48	0.076	12
Brown et al. [126]	2015	USA	Mixed	Medulloblastoma	PB	Illumina SNP chip	3	15	8	26	18	18	9	45	0.40	0.264	5
Jablonska et al. [127]	2015	Polish	Caucasian	Breast	PB	Real-time PCR	32	75	29	136	41	92	50	183	0.48	0.915	10
Parlaktas et al. [128]	2015	Turkey	Caucasian	Prostate	HB	Probe	23	23	3	49	24	20	5	49	0.31	0.784	7
Su et al. [129]	2015	China	Asian	HCC	HB	PCR-RFLP	334	78	10	422	359	107	13	479	0.14	0.150	7

MAF: minor allele frequency; HWE: Hardy-Weinberg equilibrium; HB: hospital-based; PB: population based; NA, not applicable; PCR-RFLP: polymorphism chain reaction-restriction fragment length polymorphism; MALDI-TOF MS: matrix-assisted laser desorption/ionization-time-of-flight mass spectrometry; PCR-SSCP: polymorphism chain reaction-single strand conformation polymorphism; APEX: arrayed primer extension; AS-PCR: allele specific-polymorphism chain reaction; MPM: malignant pleural mesothelioma; CRC: colorectal cancer; NHL: non-Hodgkin's lymphoma; HCC: hepatocellular carcinoma; RCC: renal cell carcinoma; OSCC: oral squamous cell carcinoma. ^a^Lin et al. [65], Tong et al. [14], and Tsai et al. [114] were only calculated for the dominant model. ^b^Cengiz et al. [15], Cheng et al. [106], and Zhang et al. [108] were only calculated for the recessive model. ^c^Ho et al. [18], Ergen et al. [79], and Atoum et al. [109] were only calculated for the heterozygous model, dominant model, and allele comparison, and the number of Ala/Ala genotype was zero. ^d^Mixed: which included more than one genotyping methods.

**Table 2:** Meta-analysis of the association between *MnSOD* Val16Ala polymorphism and cancer risk.

Variables	Number of studies	Sample size (case/controls)	Homozygous		Heterozygous		Recessive		Dominant		Allele comparison	
			Ala/Ala versus Val/Val		Val/Ala versus Val/Val		Ala/Ala versus (Val/Val + Val/Ala)		(Ala/Ala + Val/Ala) versus Val/Val		Ala versus Val	
			OR (95% CI)	*P * ^het^	OR (95% CI)	*P * ^het^	OR (95% CI)	*P * ^het^	OR (95% CI)	*P * ^het^	OR (95% CI)	*P * ^het^
All	88	33,098/37,831	**1.09 (1.00–1.19)**	<0.001	**1.07 (1.02–1.12)**	0.001	1.05 (0.99–1.11)	<0.001	**1.08 (1.02–1.14)**	<0.001	**1.06 (1.02–1.11)**	<0.001
Cancer type												
Breast	24	12,479/12,603	1.03 (0.95–1.13)	0.276	1.02 (0.96–1.09)	0.302	1.02 (0.94–1.10)	0.157	1.01 (0.94–1.09)	0.066	1.02 (0.97–1.06)	0.226
Prostate	17	7101/9146	1.04 (0.87–1.24)	0.002	**1.14 (1.05–1.24)**	0.765	1.03 (0.94–1.14)	0.225	**1.14 (1.05–1.23)**	0.552	**1.07 (1.00–1.15)**	0.106
Lung	6	2021/2347	1.13 (0.63–2.04)	0.019	1.05 (0.76–1.46)	0.016	0.91 (0.72–1.14)	0.313	1.02 (0.78–1.32)	0.021	0.98 (0.80–1.21)	0.039
Bladder	6	1271/1270	0.66 (0.39–1.13)	0.002	0.91 (0.68–1.23)	0.049	1.01 (0.83–1.24)	0.520	0.93 (0.68–1.26)	0.021	0.97 (0.80–1.19)	0.033
Pancreatic	6	1422/2043	1.01 (0.59–1.73)	0.007	1.07 (0.77–1.49)	0.032	1.08 (0.77–1.50)	0.020	1.04 (0.70–1.55)	0.002	1.04 (0.76–1.43)	<0.001
CRC	5	2258/2180	1.02 (0.86–1.20)	0.856	1.04 (0.90–1.20)	0.733	0.99 (0.86–1.13)	0.967	1.03 (0.90–1.18)	0.733	1.01 (0.93–1.09)	0.863
Ovarian	3	716,1358	1.10 (0.85–1.42)	0.839	1.15 (0.92–1.45)	0.773	1.00 (0.81–1.23)	0.973	1.13 (0.92–1.40)	0.748	1.05 (0.92–1.19)	0.836
HCC	3	593/759	1.92 (0.85–4.36)	0.050	1.15 (0.66–2.00)	0.055	1.70 (0.97–2.97)	0.162	1.36 (0.67–2.76)	0.005	1.34 (0.76–2.35)	0.001
NHL	3	2123/2479	1.96 (0.96–4.00)	<0.001	1.03 (0.89–1.19)	0.551	1.08 (0.94–1.24)	0.357	1.05 (0.92–1.20)	0.831	1.05 (0.96–1.14)	0.849
Other cancers	15	3114/3646	1.79 (1.18–2.70)	<0.001	1.25 (1.05–1.49)	0.058	1.54 (1.07–2.20)	<0.001	1.32 (1.08–1.61)	0.001	1.32 (1.08–1.61)	<0.001
Ethnicity												
Asian	18	5092/5748	**1.82 (1.15–2.88)**	0.020	1.10 (0.94–1.30)	0.001	**1.76 (1.16–2.68)**	0.065	1.08 (0.91–1.29)	<0.001	1.16 (0.96–1.40)	<0.001
Caucasian	56	23,738/26,121	1.03 (0.94–1.12)	<0.001	**1.08 (1.03–1.13)**	0.208	1.02 (0.96–1.08)	0.005	**1.08 (1.02–1.14)**	0.011	**1.04 (1.00–1.09)**	<0.001
African	7	1530/1758	1.58 (0.85–2.93)	<0.001	0.95 (0.80–1.12)	0.442	0.98 (0.79–1.21)	0.314	0.99 (0.81–1.20)	0.289	1.01 (0.87–1.17)	0.168
Mixed	7	2738/4204	1.11 (0.88–1.42)	0.141	0.98 (0.81–1.19)	0.196	1.12 (0.97–1.31)	0.187	1.02 (0.85–1.23)	0.177	1.06 (0.94–1.21)	0.107
Source of control												
PB	48	23,004/27,193	**1.10 (1.01–1.19)**	<0.001	**1.07 (1.02–1.12)**	0.263	1.02 (0.97–1.08)	0.071	**1.07 (1.02–1.13)**	0.071	**1.04 (1.00–1.08)**	0.006
HB	40	10,094/10,638	1.09 (0.88–1.35)	<0.001	1.08 (0.98–1.20)	0.003	**1.16 (1.01–1.34)**	<0.001	1.10 (0.98–1.23)	<0.001	**1.13 (1.03–1.24)**	<0.001
Quality score												
Low	39	7625/7608	1.15 (0.90–1.46)	<0.001	1.09 (0.98–1.22)	0.025	1.13 (0.99–1.29)	0.015	1.11 (0.98–1.26)	<0.001	**1.12 (1.02–1.23)**	<0.001
High	49	25,473/30,223	**1.08 (1.00–1.17)**	0.001	**1.07 (1.02–1.13)**	0.067	1.03 (0.97–1.09)	0.002	**1.07 (1.02–1.14)**	0.002	**1.04 (1.00–1.09)**	<0.001

Het: heterogeneity; CRC: colorectal cancer; HCC: hepatocellular carcinoma; NHL: non-Hodgkin's lymphoma; PB: population-based; HB: hospital-based.

**Table 3:** False-positive report probability values for associations between cancer risk and *MnSOD* Val16Ala polymorphism.

Genotype	Crude OR (95% CI)	*P* value^a^	Statistical power^b^	Prior probability				
				0.25	0.1	0.01	0.001	0.0001
All								
Homozygous	1.09 (1.00–1.19)	0.054	1.000	**0.140**	0.328	0.843	0.982	0.998
Heterozygous	1.07 (1.02–1.12)	0.004	1.000	**0.011**	**0.032**	0.267	0.787	0.974
Dominant	1.08 (1.02–1.14)	0.005	1.000	**0.016**	**0.045**	0.343	0.840	0.981
Allele comparison	1.06 (1.02–1.11)	0.013	1.000	**0.038**	**0.106**	0.567	0.930	0.992
Cancer type—prostate cancer								
Heterozygous	1.14 (1.05–1.24)	0.002	1.000	**0.007**	**0.020**	**0.183**	0.693	0.958
Dominant	1.14 (1.05–1.23)	0.001	1.000	**0.002**	**0.006**	**0.067**	0.420	0.879
Allele comparison	1.07 (1.00–1.15)	0.066	1.000	**0.165**	0.372	0.867	0.985	0.998
Ethnicity—Asian								
Homozygous	1.82 (1.15–2.88)	0.011	0.204	**0.134**	0.317	0.836	0.981	0.998
Recessive	1.76 (1.16–2.68)	0.008	0.228	**0.100**	0.249	0.785	0.974	0.997
Ethnicity–Caucasian								
Heterozygous	1.08 (1.03–1.13)	0.001	1.000	**0.003**	**0.008**	**0.078**	0.462	0.896
Dominant	1.08 (1.02–1.14)	0.005	1.000	**0.016**	**0.045**	0.343	0.840	0.981
Allele comparison	1.04 (1.00–1.09)	0.102	1.000	0.234	0.478	0.910	0.990	0.999
Control source—PB								
Homozygous	1.10 (1.01–1.19)	0.018	1.000	**0.050**	**0.136**	0.634	0.946	0.994
Heterozygous	1.07 (1.02–1.12)	0.004	1.000	**0.011**	**0.032**	0.267	0.787	0.974
Dominant	1.07 (1.02–1.13)	0.015	1.000	**0.043**	**0.119**	0.599	0.938	0.993
Allele comparison	1.04 (1.00–1.08)	0.042	1.000	**0.111**	0.273	0.805	0.977	0.998
Control source—HB								
Recessive	1.16 (1.01–1.34)	0.044	1.000	**0.116**	0.282	0.812	0.978	0.998
Allele comparison	1.13 (1.03–1.24)	0.010	1.000	**0.029**	**0.082**	0.495	0.908	0.990
Quality score—low								
Allele comparison	1.12 (1.02–1.23)	0.018	1.000	**0.051**	**0.138**	0.637	0.947	0.994
Quality score—high								
Homozygous	1.08 (1.00–1.17)	0.059	1.000	**0.151**	0.349	0.855	0.983	0.998
Heterozygous	1.07 (1.02–1.13)	0.015	1.000	**0.043**	**0.119**	0.599	0.938	0.993
Dominant	1.07 (1.02–1.14)	0.036	1.000	**0.098**	0.247	0.783	0.973	0.997
Allele comparison	1.04 (1.00–1.09)	0.102	1.000	0.234	0.478	0.910	0.990	0.999

^a^Chi-square test was used to calculate the genotype frequency distributions; ^b^statistical power was calculated using the number of observations in the subgroup and the OR and *P* values in this table.
